# Tapping Diversity From the Wild: From Sampling to Implementation

**DOI:** 10.3389/fpls.2021.626565

**Published:** 2021-01-27

**Authors:** Sariel Hübner, Michael B. Kantar

**Affiliations:** ^1^Galilee Research Institute (MIGAL), Tel-Hai College, Qiryat Shemona, Israel; ^2^Department of Tropical Plant and Soil Sciences, University of Hawai’i at Mânoa, Honolulu, HI, United States

**Keywords:** crop wild relative, genetic drag, sampling design, introgression, breeding

## Abstract

The diversity observed among crop wild relatives (CWRs) and their ability to flourish in unfavorable and harsh environments have drawn the attention of plant scientists and breeders for many decades. However, it is also recognized that the benefit gained from using CWRs in breeding is a potential rose between thorns of detrimental genetic variation that is linked to the trait of interest. Despite the increased interest in CWRs, little attention was given so far to the statistical, analytical, and technical considerations that should guide the sampling design, the germplasm characterization, and later its implementation in breeding. Here, we review the entire process of sampling and identifying beneficial genetic variation in CWRs and the challenge of using it in breeding. The ability to detect beneficial genetic variation in CWRs is strongly affected by the sampling design which should be adjusted to the spatial and temporal variation of the target species, the trait of interest, and the analytical approach used. Moreover, linkage disequilibrium is a key factor that constrains the resolution of searching for beneficial alleles along the genome, and later, the ability to deplete linked deleterious genetic variation as a consequence of genetic drag. We also discuss how technological advances in genomics, phenomics, biotechnology, and data science can improve the ability to identify beneficial genetic variation in CWRs and to exploit it in strive for higher-yielding and sustainable crops.

## Introduction

### Crop Wild Relatives—What Are They and What Benefit Do They Hold?

More is expected from the world’s food systems than at any previous time in human history (Godfray et al., 2010). The demands for food, fiber, fuel, and ecosystem services are increasing while climate perturbations are challenging agriculture production and geopolitical stability. Future projections models predict that major crops (e.g., maize, rice, wheat, soybean, and sunflower) will show increased vulnerability to these changes in many parts around the globe (Myers et al., 2017). Decreases in yield have already been reported (Ray et al., 2019) and are expected to escalate and narrow the extent of areas suitable for specific crops (Pironon et al., 2019). These changes can lead to the transformation of land uses and in extreme cases to the abandonment of previously cultivated regions (Estoque et al., 2019).

Humans have domesticated hundreds of plant species over the millennia, transforming wild forms into a domesticate by fixing traits of importance in the agricultural system (Meyer et al., 2012; Purugganan, 2019). Over time, the domesticates have diverged remarkably from the wild form through a continuous process of selection for specific features while neglecting other adaptive traits. Crop wild relatives (CWRs) are generally defined as wild species that have some level of inter-fertility with a crop (Harlan and de Wet, 1971). While some of the domesticated plants have a multitude of cross-compatible congener wild species (e.g., sunflower; Kantar et al., 2015), others have very few (e.g., chickpea, fava bean, and quinoa; Castañeda-Álvarez et al., 2016). Historically, attempts to create a standard classification for CWR relied mostly on empirical crossing experiments (Harlan and de Wet, 1971) and resulted in four main germplasm categories: primary (no crossing barriers), secondary (mild crossing barriers), tertiary (requires special techniques such as embryo rescue), and quaternary (genetic engineering technics are required). Recent CWR germplasm classifications also consider the taxonomic information (Maxted et al., 2006) and evolutionary relationships (Miller and Khoury, 2018) when the knowledge on the crossing compatibility is limited. These classifications have helped to create strategies to prioritize germplasm collection efforts around the globe.

Subsequently, one of the major limitations of using CWR in breeding is hybridization barriers between undomesticated germplasm and the crop which increases along with evolutionary divergence (Viruel et al., 2020). Usually, crossing between the crop and the primary gene pool is the most convenient and allows to create large populations for selection. In many cases, the direct ancestral species of the crop occurs in a wide range of environments and holds ample genetic diversity to be explored for advantageous alleles (Anderson et al., 2016). However, even when hybridization between the wild and the domesticated forms is straightforward, genetic drag due to limited recombination may require a very long and costly process of purging linked and detrimental genetic variation. This process involves several generations of backcrossing to the cultivated parent over a long period when molecular markers are not available. The use of molecular markers can significantly accelerate this process (Iftekharuddaula et al., 2011), provided that the beneficial alleles were indeed identified in an available wild germplasm collection with high precision and confidence. For example, the use of molecular markers in maize breeding through a backcrossing scheme generated a benefit of over 130,000 US$ compared with a conventional phenotypic scheme (Morris et al., 2003).

### Importance of Genebanks

Wild germplasm that has been collected and conserved over the last century is already available in genebanks (Byrne et al., 2018). However, these germplasm repositories often lack specific geographic information, phenotypic characterization, and indications of disease and other stress resistances. Moreover, these collections represent a snapshot of allele frequencies resulting from the preceding environmental conditions to the time of collection. This incomplete information necessitates continued and increased efforts in CWR collection across the globe. With the advent of technology, it is now possible to identify beneficial genetic variation across space and time with more precision and allow genebanks to manage collections and reduce redundancy more efficiently (Milner et al., 2019).

A new CWR germplasm collection expedition usually begins with exploring the distribution of the species of interest. Nowadays, this information is available electronically which allows obtaining detailed geo-ecological information for a first survey (e.g., www.gbif.org and www.genesys-pgr.org). Moreover, performing species distribution model analysis based on observations of existing populations can provide indications also for potential unobserved occurrence of populations across the studied area (Williams et al., 2009). Among the most successful methods to identify potential regions where unknown populations may exist for collection and conservation is the gap analysis (Box 1) which has been used to explore the distribution of the most important CWR (Castañeda-Álvarez et al., 2016; Khoury et al., 2020). Despite its limitations, this approach has been used to identify species that are in dire need of conservation, identify geographic regions that may hold unknown populations, and gather information for landscape genomic studies (reviewed in Bragg et al., 2015). Sampling germplasm based on gap analysis and using this information in landscape genomics provides a powerful approach to identify alleles that are likely responsible for adaptation to abiotic stress (Table 1). However, this approach is usually constrained by the resolution of sampling and the associated information available on climate, soil, and metadata at each sampling location. Another potentially efficient method (see caveats below) for identifying beneficial genetic variation in CWR is focused identification of germplasm strategy (FIGS), which model environmental variables of known collection locations rather than formal species distribution modeling to locate populations of potential interest (Khazaei et al., 2013).

**Box 1. Definitions of terms.**

**Table:** 

***Term***	***Definition***
Gap analysis	A method to evaluate the representation of biodiversity in conservation repositories such as genebanks. This approach assists in prioritizing efforts to collect and conserve biodiversity by identifying species that are underrepresented in genebanks collections (*in situ* conservation) or geographic regions that were not thoroughly sampled (*ex situ* analysis). Gap analysis is mainly conducted for crop wild relatives due to their applied importance.
Linkage disequilibrium	Association between alleles at two or more loci leading to higher frequency of dependence in segregation. Linkage disequilibrium is pronounced in elite domesticated germplasm but is also observed in natural wild populations due to demographic and selective constraints. The level of LD determines the resolution of mapping trait of interest in a segregating population or diversity panel.
Introgression	Transmission of genetic variation between individuals. Introgression relies on the crossing potential between the two individuals and also on the recombination landscape around the target genomic region.
Genetic drag	The negative effects of linked genetic variation to the trait of interest. Introgression of genetic variation from one source (e.g., crop wild relative) into a recipient variety involves a crossing step followed by consecutive backcrossing steps to recruit back the recipient variety properties. Linkage disequilibrium between the targeted beneficial trait for introgression and a deleterious or unfavorable genetic variation may “drag” the negative component and reduce the fitness of the hybrid. Strong linkage between the beneficial and negative genetic variation will require much more effort to purge the “dragged” negative effect.
Environmental stressor	Any environmental factor that can have a negative impact on the plant fitness. The environmental stress can be abiotic like drought and heat or biotic like disease or competition with other organisms. In many cases, stressors are correlated (abiotic and biotic) and their impact on the plant fitness is complex, thus the underlying resistance or tolerance to stress may be a convolution of multiple mechanisms.

**TABLE 1:** Studies that have identified alleles associated with abiotic stress in CWR.

Species	Stress	Gene	Sampling design	Breeding strategy	References
*Glycine soja*	Drought/heat	Glyma.08g298200	Random sampling	Marker assisted backcrossing	Anderson et al., 2016
*Glycine max*	Drought/heat	Glyma.15g127700	Random sampling	Marker assisted backcrossing	Bandillo et al., 2017
*Glycine max*	Annual precipitation	*Glyma.15G196500*	Random sampling	Marker assisted backcrossing	Li et al., 2019
*Picea glauca and Picea engelmannii*	Temperature/aridity	69_753	Random sampling	N/A	De La Torre et al., 2014
*Quercus lobata*	Temperature/osmotic stress	Contig code name 42 and 57	Transect	N/A	Sork et al., 2016
*Pinus taeda*	Aridity, temperature	EST contig 0–12 076n	Random sampling	N/A	Eckert et al., 2010
*Triticum urartu*	Temperature, precipitation, altitude	No specific gene	Random sampling	Marker assisted backcrossing	Brunazzi et al., 2018
*Medicago truncatula*	Temperature, salinity, drought, precipitation	Medtr3g029620	Random sampling	N/A	Guerrero et al., 2018
*Zea mays* ssp. *Parviglumis* and *Zea. mays* ssp. *mexicana*	Temperature, precipitation	Genomic region	Transect	N/A	Pyhäjärvi et al., 2013
*Zea mays* ssp. *Parviglumis* and *Zea. mays* ssp. *mexicana*	Niche suitability	GRMZM2G164358	Transect	N/A	Aguirre-Liguori et al., 2017
*Hordeum vulgare*	Temperature	HvPRR59	Random sampling	Marker assisted backcrossing, genomic selection	Russell et al., 2016
*Sorghum bicolor*	Climate zones	Genomic region	Random sampling	Marker assisted backcrossing	Morris et al., 2013
*Arabadopsis thaliana*	Temperature	AT1G18140	Paired sampling	While this is not a crop, the genes have become targets of selection in other crops (e.g., pennycress)	Fournier-Level et al., 2011
*Medicago truncatula*	Temperature, precipitation	7g074620.1	Random sampling	N/A	Yoder et al., 2014
*Phaseolus vulgaris*	Drought	Phvul.004G102800	Random sampling	Marker assisted backcrossing	Ariani and Gepts, 2019
*Phaseolus vulgaris*	Temperature, precipitation	*PvPdh1*	Random sampling	Marker assisted selection	Parker et al., 2019
*Oryza sativa*	Temperature	Genomic region	Cluster sampling	N/A	Qiu et al., 2017
*Oryza glaberrima*	Salt, temperature	OsHAK5	Random sampling	Marker assisted selection	Meyer et al., 2016
*Platycladus orientalis*	Ecological niche	Genomic regions	Cluster sampling	N/A	Jia et al., 2019
*Triticum aestivum*	Temperature	Genomic regions	Random sampling	Marker assisted selection/genomic selection	He et al., 2019
*Eucalyptus microcarpa*	Land use change	Genomic regions	Cluster sampling	N/A	Jordan et al., 2016
*Mangifera* spp.	Difference from cultivated	Genomic regions	Random SAMPLING	N/A	Wang et al., 2020
*Solanum pimpinellifolium*	Salt stress	Genomic regions and candidate genes	Transect sampling	Marker assisted selection	Gibson and Moyle, 2020
*Cajanus cajan*	Heat shock and disease resistance	Candidate genes	Random sampling	N/A	Rathinam et al., 2019
*Triticum* spp.	Agronomic Genes	Genomic regions and candidate genes	Random sampling	Marker assisted selection	He et al., 2019
*Vitis vinifera*	Difference from cultivated	Genomic regions	Random sampling	N/A	Migicovsky et al., 2016
*Populus balsamifera*	Climate niche	Genomic regions and structural variants	Transect sampling	N/A	Chhatre et al., 2019
*Beta* and *Patellifolia* spp.	Abiotic stress	Genomic regions	Random sampling	N/A	Monteiro et al., 2018
*Oryza rufipogon*	Abiotic stress	Genomic regions	Random sampling	Marker assisted selection	Li et al., 2020
*Cicer reticulatum Ladiz*	Cold tolerance	Genomic regions	Targeted sampling	Marker assisted selection	Mugabe et al., 2019

### Plant Collections Under the Nagoya Protocol

To create a more equitable system for biological material sharing across the globe, several international agreements were established in the past decades. The convention of biological diversity (CBD) has been the main instrument to regulate and ensure national sovereignty over biological resources including propagation material of CWRs in contrast to the previous situation of free access and sharing (McCluskey et al., 2017). The CBD regulations took a turn in 2014 with the establishment of the Nagoya protocol which provides standard guidelines for the implementation of the CBD regulations regardless of if the country, where collections are made, has ratified it. To ensure that benefits from the use of a biological resource are shared with the providing countries, the Nagoya protocol requires that agreement between the relevant authorities of the provider and the user is in place. Although these regulations seem conceptually fair, they pose some difficulties that disrupt the collection and share of CWR germplasm collections. One difficulty is the ability to obtain permits from the authorities in some countries, especially in regions of civil unrest where some collection gaps of important CWR were identified. Many times, the only accessible resource for genetic material is from historical collections preserved by different organizations around the world, yet the Nagoya protocol is vague regarding the share of historical germplasm collections; thus, the interpretation of different countries and organizations may be inconsistent and prevent sharing or use of highly beneficial material (Sherman and Henry, 2020). Germplasm collections that represent the distribution range of a species frequently cross the country jurisdiction, thus the bureaucratic burden involved in using a full germplasm collection may eventually prevent its implementation. Thus, the Nagoya protocol may in practice increase interest in genebank collections despite their caveats (see above) instead of establishing new, more traceable, and relevant collections. Finally, the effectiveness of the Nagoya protocol in the era of genome editing where actual crossing could be avoided is questionable, thus it seems that some adjustments to the regulations of biological material sharing are necessary in order to reach a balanced platform while maintaining the sovereignty of countries over their national resources.

### Using Crop Wild Relatives in Breeding

Despite the wide phenotypic diversity observed among crop varieties, the majority of genetic diversity found in the wild did not pass the genetic bottleneck of domestication or was eroded later in strive for higher yields during the improvement phase. CWRs hold many benefits for breeding especially through the reintroduction of lost genetic diversity and new adaptive alleles that can increase crop production (Zamir, 2001; Hajjar and Hodgkin, 2007; Dempewolf et al., 2017). This genetic diversity is well recognized for its value in enhancing crop resilience to stress such as disease and drought and recently also for increasing yield and nutritional value (Khoury et al., 2015). For example, there has been a long history of using CWR to bring resistance genes into cultivated germplasm (Qi et al., 2016, 2019; Zhang et al., 2016; Singh, 2019), but also for improving nutritional value (Pfeiffer and McClafferty, 2007; Khoury et al., 2015; Syfert et al., 2016) and increasing yield components (Xiao et al., 1996; Gur and Zamir, 2004; Fernie et al., 2006). This approach has proven successful in many crops (Table 1) including major crops like maize and sunflower, where hundreds of lines harboring allele introgressions from CWR were released over the years (Warburton et al., 2017). While in some cases there have not been specific loci identified through introgression, many species have used wide-hybridization for crop improvement (Migicovsky and Myles, 2017). The strategy of using germplasm collections to improve cultivated plants has a long history and the use of molecular genetic information has been quite fruitful in both annual (Tanksley and McCouch, 1997; Gayacharan et al., 2020; Raubach et al., 2020) and perennial species (Aljane et al., 2018; Marcotuli et al., 2019; Migicovsky et al., 2019; Warschefsky and von Wettberg, 2019; Wang et al., 2020). Hence, CWRs have a tremendous value for plant breeding, with an annual impact of over 100 billion dollars estimated across global agriculture (Pimentel et al., 1997). Most efforts in exploiting CWR in breeding were dedicated to enhancing disease resistance and much less to enhance adaptation to abiotic stress (Dempewolf et al., 2017). However, the environment is constantly changing and breeding must respond at the same pace of change. Thus, identification and characterization of adaptive alleles in CWR is a prolonged process that needs to be framed efficiently using the power of genomics, phenomics, and advanced approaches in data analysis.

## Study Design, Sampling, and Analysis

The ultimate goal of collecting and exploring CWR is to identify genetic variants that contribute to adaptation in natural environments and can also be beneficial in agricultural systems. Thus, the motivation in this context is mainly to identify genes and alleles with a potential benefit for crop breeding. Despite the contextual difference from ecological and evolutionary perspectives, the considerations, methodologies, and principles are akin. Generally, two types of approaches could be used to identify genes of interest in CWR: phenotype-independent approaches, such as genome scans (Lotterhos and Whitlock, 2015) and genome–environment association (GEA) analysis (Rellstab et al., 2015), and phenotype-dependent approaches including genome-wide association studies (GWAS) and QTL mapping (Stinchcombe and Hoekstra, 2008). Both types of approaches benefit from high-resolution genomic data and careful sampling design, yet each type of approach has its advantages and drawbacks that should be contemplated when searching for beneficial genetic variation in CWR.

### Sampling Designs

**FIGURE 1:**
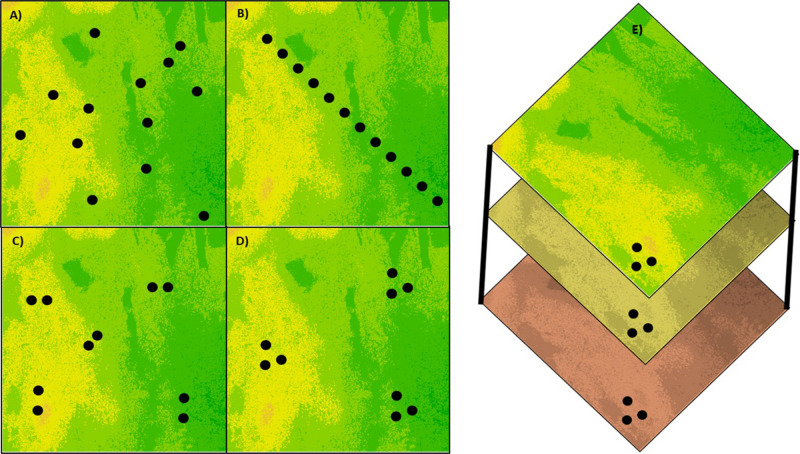
Types of sampling designs for creating CWR germplasm collections. **(A)** Random sampling to obtain an uniform and unbiased representation of a heterogeneous environment, **(B)** transect sampling to represent the variation along an environmental gradient, **(C)** paired sampling in a heterogeneous environment to reduce the demographic effect on differentiation between populations, **(D)** clustered sampling to represent the within-site variation in comparison between sites, and **(E)** sampling at the same geographic location over different seasons or time points to represent changes in allele frequencies over time.

Sampling designs impact all approaches attempting to identify beneficial genetic variation and therefore should be given considerable attention and planning (Selmoni et al., 2020). The sampling design should be adjusted according to the questions and objectives of the study (Figure 1) and the biology of the target species. One of the major confounding factors affecting the ability to identify beneficial genetic variation is the historical demography of the target species or population. This includes events of population expansion, genetic bottlenecks, recolonization, the mating strategy, and so forth. The demographic history of the species may lead to erroneous results due to a violation of the analytical model used to identify adaptive genetic variation. Moreover, the demographic history of a species is frequently correlated with environmental variation, thus distinguishing between the contribution of kinship and selective sweep to the genetic makeup may be difficult (Wright and Gaut, 2005). In the context of exploiting genetic variation obtained from CWR the objective is clear, identify genes and alleles that can enhance adaptation in crop species. The challenge is how to balance sampling across the species ecological variation to increase the chance of discovering precious genetic variation and avoid the confounding effect of past demography (Hoban et al., 2018). Different sampling strategies were examined and compared theoretically and empirically (Franco-Duran et al., 2019). Among the common strategies are transect sampling which allows representing genetic variation along ecological gradients, spatial random sampling which allows obtaining a balanced representation of the distribution range, structured sampling in demes and polygons which emphasizes the environmental variation within the distribution range, and paired-populations sampling which allows overcoming, in some cases, the confounding effects of shared demographic history (Hoban et al., 2016).

A key consideration in the sampling strategy is the number of genotypes that should be sampled to facilitate the identification of adaptive genetic variation. This is a probabilistic problem; more sampling will increase the chances of detecting beneficial genetic variation albeit with additional cost. Formerly, genotyping was the main limiting factor for sample size; however, advent in genomic sequencing technologies has made ultra-high throughput genotyping accessible; hence, genotyping a large number of individuals is now affordable (Jaworski et al., 2020). The next-generation sequencing revolution has made genome scans and GEA methods highly attractive for identifying beneficial genetic variation while genomic mapping approaches that also require high-quality phenotypic data were faced with a quickly expanding genotype–phenotype gap (Table 2). This gap is now being filled with high-throughput phenotypic data that are generated in advanced infrastructures where plants are screened and measured continuously, yet approaches for screening plants under field conditions are still rather limited and should receive more attention (Yang et al., 2020).

**TABLE 2:** Methods and software for identifying beneficial genetic variation in crop wild relatives.

Approach	Method	Statistics/strategy	Tool	References
Phenotype-independent approach	Genome scan	Standard population genetics statistics and kNN-based scans	popGenome	Pfeifer et al., 2014
		PCA-based scans for selection	pcadapt	Privé et al., 2020
		Composite test for selective sweeps	RAiSD	Alachiotis and Pavlidis, 2018
		Supervised machine learning	diploS/HIC	Kern and Schrider, 2018
		Convolutional neural network	ImaGene	Torada et al., 2019
	Genome–environment association	Fst-based genome scan	BayeScEnv	De Villemereuil and Gaggiotti, 2015
		Latent factor mixed models	LFMM2	Caye et al., 2019
		Bayesian framework for allele frequencies—environment correlation	Bayenv2	Günther and Coop, 2013
Phenotype-dependent approach	QTL mapping	Different models to map QTLS, implemented in R	R/qtl	Broman et al., 2003
		QTL mapping tool in polyploids	GWASploy	Rosyara et al., 2016
		Pooled data, univariate and multivariate QTL mapping	MultiQTL	MultiQTL Ltd., www.multiQTL.com
	Genome-wide association studies	Not requiring a reference genome	KmerGWAS	Voichek and Weigel, 2020
		GAPIT	GAPIT	Tang et al., 2016
		Univariate, multivariate, and sparse Bayesian linear mixed models	GEMMA	Zhou and Stephens, 2014
	Expression analysis	Now include a full pipeline to analyze RNA-Seq data	RSEM	Original paper: Li and Dewey, 2011
		Web-based tool to integrate results of different expression analysis packages	IDEAMEX	Jiménez-Jacinto et al., 2019

Environmental heterogeneity along space requires that sampling of individuals will properly represent the ecological gradient across the distribution range including the extremes. Occasionally, substantial adaptive genetic variation can be obtained from a single sampling location where micro-climatic conditions result in a range of environmental stressors within the same population. Moreover, theory predicts that adaptive genetic variation could also be obtained across different time points because non-random dispersion will lead to some level of environmental heterogeneity (Lynch and Ho, 2020). Thus, sampling a population without considering the site spatial and temporal variation may fail to represent the available adaptive genetic variation and introduce some bias at the exact location and timing of sampling. A tempting strategy is to sample a few populations where a high chance to identify genetic variation that is contributing to the trait of interest is expected (e.g., FIGS; Khazaei et al., 2013). Although expedient, this approach has several drawbacks. For example, sampling along a narrow geographic range can increase the rate of false-positive signals due to increased relatedness among neighboring populations. The limited geographic range and ecological variation represented may deteriorate the effectiveness of GEA and genome scans approaches. However, if enough genetic variation could be sampled, genome mapping approaches may gain power from a targeted sampling design due to the reduced effect of population structure.

Clearly, the sampling strategy and the subsequent analytical approach should be planned with much attention to the ecological and genetic characteristics of the target species. While abundant species can benefit from sampling across a spectrum of environmental gradients, species that are characterized by a constrained distribution may require an adjusted strategy of temporal repeated sampling or fine-resolution sampling of microenvironments to allow exploring the breadth of genetic variation.

### Analytical Approaches to Identify Beneficial Genetic Variation

Implementation of beneficial genetic variation obtained from CWR in breeding often requires identifying the causative mutation or tightly linked polymorphism to the trait of interest in the wild germplasm. Analytical approaches to identify the genetic variation of interest can be divided between approaches where phenotypic data are not mandatory (phenotype-independent) and approaches that rely on high-quality phenotypic data (phenotype-dependent). A short list of tools and packages is provided as an example in Table 2.

The phenotype-independent approaches do not consider the underlying trait of interest explicitly and allow to avoid laborious phenotyping experiments. This has the advantage of gaining statistical power by screening a large number of individuals and populations for signs of adaptation along the genome. Advent in genome sequencing platforms makes high-resolution genotyping for a large number of individuals accessible in a reasonable time frame and cost (Halewood et al., 2018; Jaworski et al., 2020), thus enough statistical power can be obtained to identify genomic regions of interest using these *a priori* genome screening approaches. Genome scan methods are conducted by calculating population genetics statistics using a sliding window frame in a target population (Table 2). The calculated statistics can indicate the level of diversity, linkage disequilibrium, skewness in the site frequency spectrum, or the level of differentiation between contrasting populations. Outlier scores at specific windows are interpreted as candidate regions where selective sweep in response to environmental stress occurred. These methods are highly prone to false-positive outliers due to violations of the underlying assumptions which are frequent in natural populations (Lotterhos and Whitlock, 2015; Hoban et al., 2016). The high rate of false-positive signals can partially be controlled by combining the scores or *p*-values obtained for different statistics (Lotterhos et al., 2017). However, a proper sampling scheme can significantly improve the power and accuracy of these methods (Lotterhos and Whitlock, 2015). For example, sampling pairs of populations from contrasting environmental conditions (e.g., dry/wet) and in different geographic regions can allow identifying overlapping adaptive genetic variation while controlling the demographic effect using genome scans between each pair and comparing the results obtained from a different geographic region.

Another approach to identifying genomic regions that contribute to adaptation across environmental gradients is a GEA. Like genome scans, this approach benefits from high-resolution genotyping and avoids the laborious phenotyping procedure. However, unlike genome scan methods, GEA also requires complementary environmental data. This type of information can now be obtained easily from international open databases (e.g., Fick and Hijmans, 2017), yet the data are usually derived from interpolations calculated across distant monitoring stations and averaged over long periods. Clearly, these data smooth over local extreme conditions and may poorly represent spatial and temporal heterogeneity in environmental conditions (Rellstab et al., 2015). In addition, GEA is strongly affected by spatial auto-correlation and the results may be falsely interpreted due to covariance with another, perhaps more important, environmental factor. For example, dry and warm conditions are frequently geographically correlated, thus signs of genomic associations along a drought gradient may also be obtained from genes that are contributing to the correlated response to heat. Other factors that increase the rate of false-positive signals in genome scans methods are also notable in GEA and include violations of the model assumptions, confounding effect of demographic processes, and ascertainment bias in polymorphism detection (Bragg et al., 2015; Rellstab et al., 2015).

The second type of approach used to identify beneficial genetic variation in CWR is genomic mapping using QTL analysis in a population that was generated from crosses, and GWAS conducted in a diversity panel (Table 2). To use these methods, a mapping population should be developed by selecting a representative panel of accessions for GWAS or by conducting crosses between individuals harboring specific features. Genomic mapping approaches are compelling because they target the genetic features that are contributing directly to the trait of interest as measured in controlled experimental design. These methods are generally powerful once the confounding effects of population structure and relatedness are controlled in the model, the multiple-testing effect on *p*-values inflation is corrected, and sufficient individuals are included (Korte and Farlow, 2013). Despite their effectiveness, these methods require intensive phenotyping which can be laborious and prone to noise, especially if conducted under field conditions. Other experimental facilities such as greenhouses and growth chambers are affected less by experimental variation but are concurrently less predictive for field conditions, specifically when strong genome–environment interactions exist (Tardieu et al., 2017).

Both GWAS and linkage mapping require high-resolution genotype and phenotype data for the mapping population. It is possible to use several crossing designs to generate a mapping population including a bi-parental cross between a wild accession and a modern cultivar and a multi-parental cross between several wild accessions and one or more domesticated types (Tanksley and McCouch, 1997; Meng et al., 2016). Although the mapping resolution obtained from crosses is usually low due to the limited number of recombination events observed over few generations, this approach has the great benefit of introducing novel genetic variation into elite material and generating the first phase toward the implementation of wild adaptive variation in breeding. Nevertheless, crossing CWR and modern cultivars can be challenging due to crossing barriers. These barriers increase with the divergence between the wild germplasm and the cultivated lines, thus crosses with the primary gene pool have the highest (or sometimes the only) chance of producing a viable offspring for subsequent implementation through breeding.

Another powerful approach to identify beneficial genetic variation in wild germplasm is conducting a differential expression analysis between wild and cultivated accessions or between wild accessions exposed to different treatments (Hübner et al., 2015). This approach has the benefit of handling a small number of accessions to focus on the trait of interest and the underlying genes. However, prior knowledge is required on the exact timing and tissue where the relevant genes are expressed and indications of the plausible identity of these genes. Otherwise, it is challenging to distinguish, based on the differential expression profile alone, between genes that truly contribute to the trait of interest and those of only mild effect (Azodi et al., 2020).

## From Survey to Implementation

### Introgression of Beneficial Traits

In many crop species, yields are starting to plateau presumably due to erosion of genetic variation (albeit there are also other reasons) that hinders adaptation to increasing environmental stress. Therefore, enhancing adaptation in elite varieties by introgression of new genetic variation from wild relatives is a promising venture. Introgression of beneficial genetic variation from CWR is not a new concept, and there have been many successful attempts to enhance adaptation mainly through increasing biotic and abiotic stress tolerance in various crop species (Hajjar and Hodgkin, 2007; Sharma and Upadhyaya, 2016; Hübner et al., 2019; Szymañski et al., 2020). Some of the well-known examples include rust resistance in wheat (Autrique et al., 1995), cytoplasmic male sterility in sunflower (Rieseberg et al., 1994), and submergence tolerance in rice (Xu et al., 2006). Nevertheless, introgression comes with a burden as linked deleterious genetic variation often accompanies the trait of interest causing a genetic drag. To achieve a successful integration of a beneficial trait while minimizing the associated genetic drag, the recombination landscape at the region of introgression should be explored. Although many introgressions from wild germplasm have resulted in a substantial non-recombining haplotype (Baute et al., 2015), it is unclear to what extent the deleterious effect of genetic drag deteriorates the performance of the recipient cultivar.

In eukaryotes, the recombination rate varies significantly across species, populations, and individuals. The recombination rate also varies across the chromosome which appears as regions of elevated recombination (hot-spots) or suppressed recombination (cold-spots) along the genome. Likewise, recombination landscape varies between crop species and their wild relatives, thus recombination hot-spots and cold-spots do not necessarily occur at overlapping positions in both species (Dreissig et al., 2019). Introgression is also affected by the recombination landscape along the genome. First, regions of homology tend to recombine more, thus the higher the homology between species the higher the chance for successful introgression (Canady et al., 2006). This correlation between homology and introgression advocates the use of the primary gene pool for identification of beneficial genetic variation and as the source of introgression, preferably from the ancestral population of the crop species, if known.

Second, introgression from a congener species through hybridization and backcrossing could be efficiently cleaved in recombination hot-spots and consequently purged by selection. Hence, the deleterious effect of genetic drag can be depleted efficiently while the beneficial allele is fixed (Blary and Jenczewski, 2019). Nevertheless, if the beneficial trait is attributed to linked alleles that are passed together to the recipient genotype, a high recombination rate at the introgression region can also break this advantageous linkage (Sachdeva and Barton, 2018). Targeting the introgression to a recombination hot-spot may be difficult and depends largely on the homology and the recombination landscape of both the recipient and donor individuals. Moreover, the introgression itself can have a dramatic effect on the recombination landscape around the introgression region in the recipient species (Rodgers-Melnick et al., 2015). In crops where double haploid production protocols are established, a genotype could be fixed at a homozygote state quickly once the introgressed region around the beneficial variant is minimized (Daurova et al., 2020). The development of high-resolution markers that are tightly linked to the causative mutation is important to track the course of introgression throughout the breeding process. Nevertheless, evidence for the contribution of structural variation such as inversions, insertions, and deletions to adaptation is accumulating in many crop species (Zhou et al., 2019; Alonge et al., 2020; Todesco et al., 2020; Walkowiak et al., 2020). Increasing the resolution of molecular markers within these regions would yield little benefit because recombination within those regions is rare.

As the ultimate goal is to be able to introgress the traits into a breeding material, sampling ecotypes that are very diverged from the cultivated gene pool may pose challenges to successful introgression of the anticipated beneficial genetic variation. Thus, identifying the progenitor wild population of the cultivated gene pool or at least the genetically closest can be critical for an efficient and successful introgression. Due to local environmental variation, beneficial alleles could potentially also be found in a genetically closer population to the cultivated gene pool (Hübner et al., 2015). The higher genetic resemblance between the crop and its wild relative can potentially reduce the genetic drag and the deleterious effect of background selection in the wild source.

Another consideration for the implementation of CWR in crop improvement is the breeding strategy of the target species. In many crops, breeders are crossing distinct inbred parents to produce a hybrid variety with increased vigor and yield. In these cases, the interaction between the introgressed wild allele and the cultivated allele at the adaptive gene should also be considered. Overdominance heterotic interactions are difficult to predict in advance and, in some cases, dominance of the cultivated allele may mask the effect of the wild allele. Nevertheless, at least some of the heterotic effects observed in hybrid crops are caused by genetic complementation from the wild parent (Owens et al., 2019), thus allowing to exploit effectively beneficial genetic variation from CWR. Another level of complexity in introgression of beneficial genetic variation from CWR is when the target species is a polyploid. The potential genome asymmetry should also be considered on top of all other factors described for diploid species. These considerations are further complicated when there are ploidy differences between the donor and recipient species (Viruel et al., 2020). Unlike annuals crops, introgression of alleles from CWR in perennial species is more challenging due to the long generation time. However, once a beneficial successful introgression is observed in breeding material, maintenance of the new variety is potentially simpler if clonal propagation is possible.

### Direct Uses of Crop Wild Relatives

Domestication is a long endeavor, which includes the fixation of many quantitative traits. Therefore, to develop a fully domesticated crop from a wild species, a long breeding process is required before all domestication syndrome traits are fixed and acceptable yields and biomass are obtained. Thus, it is not surprising that only a few plant species went through a complete process of domestication to meet modern agriculture standards (Stetter et al., 2017). Among the successfully domesticated species are the 15 major crops cultivated today around the globe, which provide circa 70% of the calories in human nutrition (Khoury et al., 2014). Interestingly, half of the calories produced globally are acquired from the three major annual crops, i.e., wheat, rice, and maize (Ladha et al., 2016). Although only a few species went through the entire process of domestication and improvement, many other species were only partially domesticated (e.g., Kiwiberry-Hale et al., 2018; Pennycress-Chopra et al., 2020). Obviously, these crops cannot compete with the high yields of the elite crops, yet they hold many nutritional and adaptive advantages as they can be cultivated in a wide range of environments with less agronomic input and lower ecological footprint (Fernie and Yan, 2019). Moreover, semi-domesticated crops are characterized by higher local adaptation at specific environments compared with elite crop varieties specifically at regions where elite cultivars have low suitability and require intensive investment to produce high yields.

Two routes of domestication can be pursued: re-domestication of a wild form of an existing domesticated crop and *de novo* domestication (e.g., the Eastern North American domestication complex; Mueller et al., 2017) of a species that has never been successfully domesticated or that has been domesticated before but for a different purpose (Fernie and Yan, 2019). Re-domestication of CWRs has the benefit of having a reference of a previous success, namely, domestication of the target species is indeed possible and the domestication syndrome traits are known. Accordingly, advanced biotechnology including transformation and gene editing is emerging as attractive techniques to target domestication syndrome traits to develop a domesticated crop (Lemmon et al., 2018). This biotechnological approach can potentially avoid the deleterious effect of genetic drag that is frequently associated with classical breeding. It is tempting to speculate that if a super-gene is targeted and modified, a more concrete domesticated phenotype could be obtained in a few laboratory steps. Another route is *de novo* domestication of a wild species that was not successfully domesticated before but has specific properties that make it commercially attractive (Fernie and Yan, 2019). Certainly, this is a long process that could span over many generations if a fully domesticated form is desirable. However, the process could be shortened significantly if directed at specific use such as the production of valuable metabolite, chemical compound, highly nutritional component, and so forth (Pinela et al., 2017). Domestication approaches are particularly useful in perennial species with long generation times. Selected wild accessions could be used as a founder breeding population and optimized through the application of genomic selection of desired and beneficial traits (Cooper et al., 2016).

## Future Directions

### Advancement in Genomics

In the last two decades, the field of genomics has made a quantum leap making genome sequencing of any organism affordable and accessible. CWRs with large and repetitive genomes can now be sequenced at large scale and reference genomes could be assembled within months (including polyploid and outcrossing species). Other levels of genetic variation, including gene inversions, deletions, insertions, and duplications, are emerging as key factors in evolutionary biology and the generation of phenotypic variation within breeding material (Hübner et al., 2019; Tao et al., 2019; Alonge et al., 2020). Thus, high-resolution genomic characterization is no longer the limiting factor in targeting beneficial genetic variation in CWR. In contrast, high-quality phenotype data for CWR that were generated under field or controlled conditions are still rather limited, but availability is increasing (Raubach et al., 2020). Setting large phenotyping experiments for wild germplasm is challenging, yet the unprecedented benefit for the scientific and breeding community should encourage more investment and attention. With the advent of technology, large phenotyping experiments become more applicable, thus data collecting and sharing standards should be established to allow accessible archiving and pulling of information through public repositories (Zamir, 2001). The recent biotechnological breakthrough of genome editing engineering toolkit such as CRISPR is expected to dramatically impact the implementation of genetic variation identified in CWR. Genome editing has the advantage of targeting precisely the genomic factors to be edited and helps to avoid many generations of backcrossing to reduce the negative effect of genetic drag and potentially also circumvent crossing barriers. Genome editing could be used to manipulate both qualitative and quantitative traits (Chen et al., 2019) following the beneficial genetic variation identified in CWR. Editing protocols involve transformation and tissue culturing steps which could be challenging in some crops. However, active research and developments may circumvent these steps and allow the implementation of this technology beyond specific genotypes and species and make it applicable more broadly (e.g., bombardment, nano-particles; Kausch et al., 2019). Moreover, in cases where the transition from a wild phenotype to a domesticate involves a small set of genes, genome editing could be used to *de novo* domesticate wild species in a short timeframe (Lemmon et al., 2018).

### Advancement in Data Collection and Analysis

**FIGURE 2:**
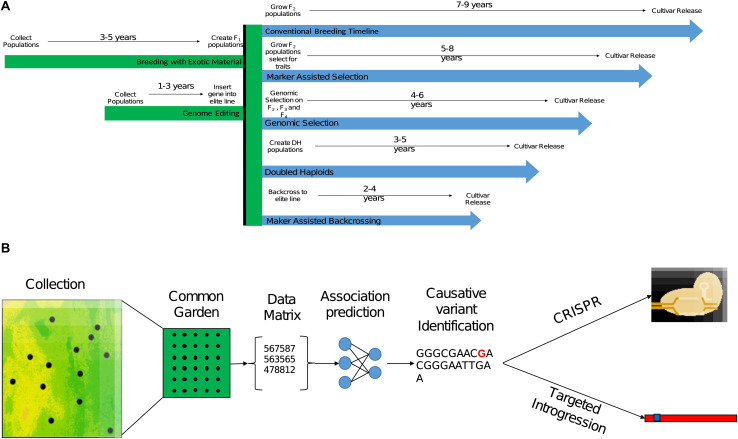
The process of implementing beneficial genetic variation identified in CWR in breeding. **(A)** Expected breeding timeline for different breeding strategies leveraging beneficial genetic variation that was already targeted in CWR. The given time frames are for annual crop breeding without the use of accelerating conditions such as greenhouse or winter nursery. **(B)** A conceptual pipeline for leveraging genetic variation identified in CWR in breeding, from sampling design and collection, followed with a common garden experiment for phenotyping, analysis of genomic, phenomic, and environmental data to target the causative mutation or tightly linked polymorphism. Once the trait was targeted gnomically, implementation into breeding material could be conducted through direct genome editing (CRISPR) or introgression.

Data collecting technologies are emerging as interesting means to monitor phenotypes in large-scale field trials. Moreover, the integration of continuous data recording instruments and remote sensing devices is now allowing researchers and breeders to monitor and perform detailed phenotyping of CWR in their natural habitats (Rebetzke et al., 2019). In the past few years, a powerful data analysis approach to leverage high dimensionality data using machine learning (ML) algorithms has gained considerable attention. Although this approach is still in its infancy in the field of quantitative and population genetics, it is quickly emerging as an accurate predicting tool that can overcome some of the unrealistic assumptions of population genetics models (Schrider and Kern, 2018). Algorithms to identify footprints of selective sweeps in natural populations and genotype–phenotype associations are becoming available for the community (Table 2). These analytical tools are expected to significantly improve the predictability of the causative mutation(s) through *post hoc* analysis especially in complex traits (Ramstein et al., 2019; Nicholls et al., 2020). Other applications of ML algorithms can help to accelerate the breeding process through the implementation of deep learning methods in phenotyping, genomic selection, prediction of functionality, and so forth (reviewed in Wang et al., 2019). Rapid technological advances in data production and analysis can facilitate the use of CWR in breeding more broadly than before (Figure 2).

Genomic and phenomic data could now be generated in large quantities and reasonable budget, thus the genotype-phenotype gap is quickly shrinking thanks to technology (Prohens et al., 2017; Yang et al., 2020). This has a substantial impact on the use of CWR in breeding because traits can be efficiently targeted, transferred, and fixed in cultivated material. Thus, identification of beneficial genetic variation in nature using ML algorithms will allow developing crop ideotypes at a much higher pace than ever before and the value of these resources will only increase over time.

## Conclusion

Crop wild relatives have long been recognized as a highly valuable resource of genetic variation that could be exploited in breeding. Many examples of successful implementation of wild genetic variation in breeding exist yet much more could be exploited to improve the nutritional value of crops, increase their resilience to biotic and abiotic stress, and enhance their economic yield. Targeting beneficial genetic variation in nature is challenging and requires a careful sampling design that considers the ecological and evolutionary properties of the target species. Advent in high-throughput genotyping technology coupled with ongoing developments in computation power and machine-learning algorithms is allowing to identify beneficial genetic variation in CWR at the finest resolution. Thus, the implementation of wild genetic variation in breeding is expected to increase in the near future thanks to the ability to narrow the introgressed region and reduce the effect of genetic drag. Genome editing technology is quickly emerging as a promising approach to introduce beneficial genetic variation and avoid some of the complications associated with crossing. Although this technology is still not fully functional in many crop species, new protocols are emerging, thus CWRs are becoming more relevant for breeding than ever before.

## Author Contributions

Both authors listed have made a substantial, direct and intellectual contribution to the work, and approved it for publication.

## Conflict of Interest

The authors declare that the research was conducted in the absence of any commercial or financial relationships that could be construed as a potential conflict of interest.
